# Interactions between CNS and immune cells in tuberculous meningitis

**DOI:** 10.3389/fimmu.2024.1326859

**Published:** 2024-02-01

**Authors:** Quan Ma, Jinyun Chen, Xingxing Kong, Yuqin Zeng, Zhanpeng Chen, Huazhen Liu, Lanlan Liu, Shuihua Lu, Xiaomin Wang

**Affiliations:** National Clinical Research Center for Infectious Disease, Shenzhen Third People's Hospital, Shenzhen, Guangdong, China

**Keywords:** central nervous system, immune cells, tuberculous meningitis, microglia, macrophages

## Abstract

The central nervous system (CNS) harbors its own special immune system composed of microglia in the parenchyma, CNS-associated macrophages (CAMs), dendritic cells, monocytes, and the barrier systems within the brain. Recently, advances in the immune cells in the CNS provided new insights to understand the development of tuberculous meningitis (TBM), which is the predominant form of *Mycobacterium tuberculosis* (*M.tb*) infection in the CNS and accompanied with high mortality and disability. The development of the CNS requires the protection of immune cells, including macrophages and microglia, during embryogenesis to ensure the accurate development of the CNS and immune response following pathogenic invasion. In this review, we summarize the current understanding on the CNS immune cells during the initiation and development of the TBM. We also explore the interactions of immune cells with the CNS in TBM. In the future, the combination of modern techniques should be applied to explore the role of immune cells of CNS in TBM.

## 1 Introduction

Tuberculosis (TB) is one of the communicable diseases and the leading causes of health problems, which causes more deaths from a single agent of infection [ranking above human immunodeficiency virus/acquired immunodeficiency syndrome (HIV/AIDS)] (1). TB patients suffered from bacillus *Mycobacterium tuberculosis* (*M.tb)* infection when exposed to air containing *M.tb* after coughing by TB patients. Although approximately a quarter of the world’s population is supposed to be infected with *M.tb*, most people will not develop TB (2). TB is the first cause of death by a single infectious agent and ranks as the 13th leading cause of death worldwide. However, the *Mycobacterium bovis* bacille Calmette–Guérin (BCG), which is the only licensed vaccine in clinical practice, is applied to prevent tuberculosis. Notably, in global TB deaths among HIV-negative or HIV-positive people, 54% or 51% were men, 32% or 38% were women, and 14% or 11% were children, respectively. In summary, approximately 90% of the TB patients are adults, with the number of men bigger than that of women in adult TB patients (3). In TB patients, *M.tb* mainly affects the lungs (pulmonary TB), and extrapulmonary sites including the central nervous system (CNS), lymph nodes, and pleura could also be affected (4–6). Diffusion of *M.tb* to the brain may induce the most severe form of extrapulmonary TB, tuberculous meningitis (TBM), which is the predominant form of *M.tb* infection in the CNS and accompanied with high mortality and disability. Moreover, TBM is the most serious extrapulmonary form of TB with high mortality up to 50% in the HIV co-infected patients (7, 8). Although the morbidity of TBM in TB patients is approximately 1%, TBM causes a higher rate of mortality and morbidity in young children compared to that in adults (9).

A retrospective, descriptive study in Beijing Children’s Hospital revealed that approximately 50% of the TB patients had extrapulmonary TB and approximately 39% had TBM in extrapulmonary TB (10). An investigation of national surveillance data of Germany from 2002 to 2009 showed that of the pediatric TB patients, approximately 3.9% of those younger than 5 years, 2.2% of those aged 5–9 years, and 1.3% of those aged 10–14 years had TBM (11). In the referral center for infectious diseases in Thessaloniki, 43 children aged 7 months to 13 years, of whom 14 children (33%) were identified as having an extrameningeal site of infection, 14 children (33%) were identified as having pulmonary tuberculosis, and 1 child was diagnosed with spondylitis, had been diagnosed with TBM from 1984 to 2008 with a gradually decreasing trend over the years (12). Despite the development and application of many methods for detecting *M.tb*, the diagnosis of TBM and some other extrapulmonary TB continues to face a huge challenge. Nicholas et al. found that a caseating focus existed in brain parenchyma or meninges in TBM patients (13). Moreover, modified Ziehl–Neelsen (ZN) staining in the cerebrospinal fluid (CSF), GeneXpert, and culture of cerebrospinal fluid were applied to improve the microbiological diagnosis of TBM (14). High levels of CSF volume and lactate and lower blood glucose ratio were independently related with microbiological confirmation of TBM. *M.tb* infects the CNS from the lungs through hematogenous transmission. Briefly, the deposition of *M.tb* in the brain is known as “Rich foci” during bacteremia in the early stage of TB. In the CNS, the “Rich foci” existed in the subpial or subependymal areas of the brain, bacilli, and meninges kept in a dormant state for a long time. The initiation of TBM was made by the growing and rupturing of those “Rich foci” into the ventricular system or subarachnoid space (15). Generally, primary infection in the brain may lead to the occurrence of TBM from 6 to 12 months (16). The *M.tb* has the potential to invade and migrate into the CNS by crossing the blood–brain barrier (BBB) and blood–CSF barrier (BCSFB) with the existence of virulence and immune factors, exocytosis, and longer intracellular survival (17, 18). Although the role of immune system in the diffusion of *M.tb* to the peripheral system has been well studied, the role of immune cells in the CNS has not been well reviewed. Here, we summarize the role of immune cells in the CNS during the progress of TBM with an aim of helping to understand the interaction between TBM and immune system in the CNS.

The brain had been regarded as “immune privileged” by wrapping with some barriers in the early twentieth century. This hypothesis had been supported by two experiments that injection of a dye in peripheral blood did not enter the brain (19) and the survival of tissue grafts was prolonged by transplantation of the brain tissue compared to transplantation of peripheral tissues (20). These may lead to a conclusion that the brain is an independent immune system isolated from the peripheral immune system. Otherwise, microglia, the major brain-resident immune cell in the brain, is considered as an argument to support the brain as an independent immune system. However, the fact that the lymphatic system had been found in the brain as early as the early twentieth century has been ignored and did not apply to oppose the “immune privilege” of the brain (21). Moreover, some adaptive immune cells have been identified in CNS in some studies that have been carried out in mammal models of neuroinflammatory diseases (22–25). The interaction between the brain and peripheral immune system has been suggested to be achieved in some mammal models, in which bone-marrow-produced T cells (26) and macrophages (27) exert a protection and repair role in the CNS. In addition, T cells play a critical role in maintaining brain function by recognizing brain self-antigens (26). Thus, these studies demonstrate that the protective autoimmunity in the CNS can enhance brain repair and maintain brain homeostasis by self-recognizing immune cells. The adaptive immune cells are thought to support the formation of new neurons in the CNS (28, 29). Thus, adaptive immune cells in the CNS are involved in social behavior (30–33), emotional stress (34, 35), and cognition (36, 37). In this review, we aim to summarize the role of immune cells in the CNS during the initiation and development of TBM and then explore the interaction of immune cells with the CNS in TBM.

## 2 Biological roles of CNS macrophages in TBM

The development of CNS requires the protection of immune cells, including macrophages and microglia, during embryogenesis to ensure the accurate development of CNS and immune response following pathogenic invasion (38, 39). Therefore, macrophages exert their immune functions in the brain very early during embryogenesis and development. The differentiation in CNS needs a highly integrated process during the embryogenesis and development by genetic and extrinsic factors. Macrophages act as the main reservoir for *M.tb*, and its survival depends on its ability to evade the killing mechanisms of macrophages (40, 41). Some macrophages infected with *M.tb* act as a “Trojan horse”, which carry *M.tb* across the BBB (42). It is difficult for the anti-tuberculosis drugs to cross the BBB and target the CNS macrophages, which cannot effectively eliminate *M.tb*, bringing a great challenge to the treatment of TBM (43).

Neuroinflammation in the CNS caused by pathogenic infections recruits macrophages and releases chemokines and cytokines in the parenchyma, which then opens the BBB (44). TBM patients who received a 7-day course of standard first-line anti-tuberculosis therapy showed excellent improvement, with increased numbers of macrophages and lymphocytes in the CSF capable of engulfing *M.tb* (45, 46). Then, the macrophages induced the host innate immunity after *M.tb* infection through inducing inflammatory response and pathogen recognition (47). Shao et al. demonstrated that detection of mycobacterial antigens in the CSF macrophages by using immunocytochemical staining had a sensitivity or specificity of 73.5% or 90.7%, respectively, and could be applied in diagnosing TBM in clinical practice (48).

The CNS macrophages exerted its protection by equipping nucleotide-binding oligomerization domain-like receptors (NLRs), PRRs, and TLRs in the CNS (49–54). Clearance of invading pathogens through phagocytosis is accompanied by the release of proinflammatory cytokines and chemokines (55), which then activate neighboring microglia and recruit other immune cells to treat this infection (56–62). The cell apoptosis or programmed cell death occurs after *M.tb* infection and then reduce the viability of mycobacteria. The level of apoptosis in alveolar macrophages after infection with attenuated *M. tuberculosis* H37Ra is higher than those with the virulent H37Rv strain infection (63). Therefore, the cell apoptosis in macrophage may exert a protective role in *M.tb* infection.

## 3 Biological roles of microglia in TBM

*M.tb*, the infectious agent of TB, replicates and propagates from the respiratory epithelium and then enters the CNS by breaking through the BBB and causes primary infection in the meninges or the brain. Microglia are resident macrophages and are major components of the brain’s immune system, working with other immune cells and neurons to maintain the brain’s homeostasis and prevent the invasion of harmful pathogens. Microglia fulfill their physiological roles throughout their lives, which is independent from the blood circle, and maintain apoptosis and proliferation under physiological conditions (64–66). Recently, studies have illustrated that microglia only originate in the erythromyeloid progenitors (EMPs) in the embryonic yolk sac (67, 68) and that microglia have a long lifespan with a low-rate clonal expansion (69, 70). Microglia are the first immune cells in the CNS when infected with *M.tb*, and the activation of microglia results in the progression of infection. However, Tucker et al. have demonstrated that microglia have been activated in a pediatric rabbit model of TBM (71). Zhou et al. showed that microRNA-124 modulates the proliferation of *Mycobacterium* by inhibiting the signal transducer and activator of transcription 3 signaling pathway (72). Although microglia play an immune supervisor role in the CNS, the long-lasting activation of microglia induces hyper-neuroinflammation, which may induce neurotoxicity and impair the cognitive ability in TB patients (73). As the main component of myeloid cells, microglia infiltration reflects the brain inflammatory activation status in TBM (74). The proinflammatory cytokines and chemokines secreted by activated microglia after the *M.tb* infection trigger neurotoxicity and tissue injuries in the CNS. Microglia have both “resting” and “activated” status, and various factors including viral infection in the peripheral nervous system and brain trauma may alter the balance between “resting” and “activated” state (75). Microglia are primarily involved in the maintenance of a “resting” state and perform kinds of functions including pathogen protection, neuron nourishment, and debris removal (76–78).

While neurons and astrocytes may be potential targets for *M.tb* infection, microglia have been considered to be the first target in the CNS due to their properties related to macrophages (79, 80). Studies have shown that human microglia and astrocytes have been intracellularly infected with *M.tb* H37Rv strain after 24 h *in vitro* treatment. Notably, most of the microglia infected with *M.tb* were infected with 4.2 bacilli per cell. However, only 15% astrocytes were infected with 1.3 bacilli per cell (80). The difference in the infection rate of *M.tb* between microglia and astrocytes may be due to the expression of CD14 receptors in microglia (81, 82).

The *M.tb* could be recognized by microglia via the innate immune and neuro-specific receptors. After CNS infection, activated microglia not only produce tumor necrosis factor-α (TNF-α) and interleukin-8 but also express an amount of immune-recognition molecules in the innate immunity (83, 84). The particular pattern recognition receptors (PRRs) in microglia are essential for initiating a fast response during the invasion of *M.tb* in microglia (85, 86). In the antigen recognition process, *M.tb* could seriously damage microglia (87). The Toll-like receptors (TLRs), intracellular PRRs, C-type lectin receptors, complement receptor 3, triggering receptors expressed on myeloid cells (TREM), and myeloid DAP-12-associated lectin (MDL-1) are a member of intracellular PRRs in microglia (88). TLR co-receptors CD14, TLR1-TLR4, and TLR5-TLR9 are mostly expressed in microglia. Furthermore, previous studies have shown that TLR1, TLR2, and TLR4 are mainly presented on the surface of microglia, whereas TLR3, TLR7, and TLR8 are predominantly located intracellularly (49, 52, 88). Although studies have found that the TLR mechanisms in neurodegenerative disorders [Alzheimer’s disease (AD), spinal cord injury, amyotrophic lateral sclerosis, and Parkinson’s disease (PD)], pathogenic infections (HIV, Japanese encephalitis virus (JEV), and *Neisseria meningitidis*), and ischemic brain injury have been well studied (89–95), the TLR mechanisms in TBM remain unclear. Previous findings revealed that the TLR4 binds to CD14 on the surface of microglia through lipopolysaccharides (LPS) in the internalization of *M.tb* (82). On the other hand, TLR2, TLR4, and TLR9 pathways activated their downstream phosphorylation and cytokine production during the internalization of *M.tb* in macrophages (96–98). Understanding the mechanism of the role of PRRs on macrophages in *M.tb* pathogenic infection and damage recognition will help to explore the mechanism of PRRs in microglia during the progress of TBM. Another report has shown that both murine microglial TLR2 and dectin-1 were not participated in inflammatory responses induced by *M.tb* (99). Consequently, the mechanism by which microglia recognize *M.tb* after infection remains unclear.

*Mycobacterium marinum* has been applied to explore the role of microglial autophagy in TBM. *Mycobacterium marinum* infection induces the formation of granulomas in the zebrafish brain. It also induces the microglial autophagy in response to its replication (100). However, the production of interleukin-6 (IL-6) from macrophages inhibits interferon-gamma (IFN-γ)-induced autophagy and then combats innate immunity after *M. marinum* infection and increases the intracellular persistence (101).

Recently, a macrophage-inducible C-type lectin (Mincle), which belongs to the C-type lectin receptor family, may be become a potential target for Mincle agonist (102). Trehalose-6,6-dibehenate (TDB), a novel adjuvant for TB vaccines and a synthetic analog of trehalose6,6-dimycolate (TDM), was applied in human clinical studies. Both TDM and TDB induce inflammatory gene expression in macrophages and dendritic cells by binding to Mincle (103, 104). Although the expression of Mincle is limited in the primary microglia, TDB reduces the TLR4-mediated neuroinflammation in Mincle-knockout microglia and mice. Notably, these results in Mincle-knockout microglia and mice differed from their performance in macrophages. However, the mechanism of TDB in modulating C-gamma 1 (PLC-γ1) signaling pathway remains unclear. The *M.tb* may persist and multiply intracellularly after being internalized by microglia (81, 105). In the development of TBM, *M.tb* leads to microglial accumulation and activation and then triggers the granulomatous formation (106, 107). Three distinct granuloma types including non-necrotizing, necrotizing gummatous, and necrotizing abscess were distinguished. All types of granulomatous were observed in each patient and mainly located in the perivascular areas of the leptomeninges. The size of non-necrotizing granuloma (0.1–0.5 mm) was smaller than the necrotizing gummatous (≥ 5 mm) and necrotizing abscess (10 mm). In TBM patients, granulomas were wrapped with a blood vessel and then damaged by vasculitis in a later stage.

Yang et al. found that inhibiting secretory phospholipase A_2_ (sPLA_2_) could attenuate the ROS and various inflammatory mediators production after *M.tb* infection in murine microglial BV-2 cells. Inhibition of the Ras/Raf-1/MEK1/ERK1/2 signaling pathway diminished sPLA_2_ activity after *M.tb* infection in BV-2 cells (108). Mitochondrial ROS, potassium efflux, and lysosomal proteases cathepsin B promote activation of nucleotide binding and oligomerization of the domain-like receptor family pyrin domain containing 3 protein (NLRP3) inflammasome activation in response to *M.tb* infection (109).

## 4 Biological roles of B and T lymphocytes in TBM

Activated T and B lymphocytes have been found in the CSF of TBM patients from the clinical onset of disease. In addition, T and B lymphocytes were involved in the adaptive cellular immune response in TBM patients (106). Meningeal B lymphocytes were present in secondary progressive multiple sclerosis (SPMS) and were responsible for inducing pathology by producing inflammatory mediators and participating in antigen presentation (110–112). Anti-CD20 may be a potential therapeutic target for meningeal B-lymphocytes aggregation (113). During the development of TBM, the humoral response was sustained for approximately 2 months and the cellular immune response was maintained for another 3 months (114). Zhang et al. illustrated that the distribution of lymphocyte subpopulations and the ratio of CD4:CD8 in CSF were different in patients with anti-N-methyl-D-aspartate receptor AE (NMDAR-AE), herpes simplex virus encephalitis (HSVE), and TBM (115). After clinical therapy of TBM, the number of CD4- and CD45RO-positive T cells increased significantly in CSF (116). Therefore, the levels of CD4 and CD45RO positive T cells were useful biomarkers for diagnosing TBM. The T lymphocytes and CD4-positive helper T (Th) cells play a key role in granuloma formation in TBM patient, and the interactions between CD4^+^ Th cells and T lymphocytes induce the IFN-γ production and then activate the macrophages to engulf and digest *M.tb* (117–120). Another report indicated that IL-2-positive T lymphocytes in the CSF of TBM patients can produce specific and reactive antigen when stimulated with IL-2, antigen-presenting cells (APC), or Muromonab CD3 (OKT3) antibody (121). Xu et al. found that the expression levels of Th1, Th2, Th 17, TNF-α, and TNF-β were elevated in TBM patients compared to those without the CNS infections in HIV-infected persons (122). Moreover, the expression levels of IFN-γ, regulated upon activation normal T cells expressed and presumably secreted (RANTES) and interferon-inducible protein (IP-10) in TBM patients were higher than in non-TBM with HIV infection. The cytokines/chemokines of Th1, Th2, and Th17 exerted a critical role in the pathogenesis of TBM. The upregulation of IL-17a, TNF-β, IL-5, IL-12p40, and IL-1Rα in CSF has been demonstrated to be related with meningitis. Thus, Kösters et al. have developed a valuable diagnostic tool for the diagnosis of neurotuberculosis including TBM by using a T-cell IFN-γ release assay in clinical practice (123). More diagnostic tools for measuring cytokines/chemokines in TBM should be developed in clinical practice.

## 5 Biological roles of neutrophil in TBM

Neutrophils do not exist in the border zone encephalitis areas of parenchyma under physiological conditions. The absence of neutrophils in the brain is due to the protection of CNS against the aggressive cells, including neutrophils, across the BBB. Neutrophils have a large number of enzymes in their membrane-bound granules that produce reactive oxygen species and thereby affect the cellular environment. In the early stage of TBM, the BBB prevents neutrophils from entering the CNS and avoids the neural damage (106). In TBM patients, the number of CSF neutrophils and *M.tb*-positive presentation were considered for the characterization of the immune reconstitution inflammatory syndrome (IRIS) (124). Additionally, CSF neutrophil mediators, including S100A8/9, were significantly increased in patients with TBM-IRIS compared to those without (125, 126). Marais et al. illustrated that neutrophil-abundant transcripts increased in the progression of developing IRIS until the onset of TBM-IRIS by performing longitudinal microarray analysis of the blood (127, 128). After the diagnosis of TBM was confirmed, TBM patients received first-line anti-tuberculosis treatment. After 7 days treatments of anti-TB, all patients showed excellent improvement, with 82% lymphocytes and macrophages in CSF participated in engulfing *M.tb*. Then, the number of neutrophils and the protein level were increased in CSF after reducing the dose of dexamethasone during the first-line therapy of anti-TB; those findings indicated a relapse of TBM. Moreover, the strong relationship between neutrophils and matrix metalloproteinase-9 (MMP-9) in CSF has been found in the TBM after dexamethasone treatment (129–131). However, a high level of neutrophils in CSF has a positive impact on the chance of survival in TBI patients and was associated with the occurrence of cerebral infarction by using head MRI in adult TBM patients (132–135). In addition, the neutrophil proportion in the CSF is independently associated with TBM, and doctors can use five indexes including neutrophil proportion, disease course, white blood cell count, serum sodium, and total white cell count in the CSF to distinguish TBM from bacterial meningitis (136–142).

## 6 Biological roles of natural killer cells in TBM

Natural killer (NK) cells are upregulated in the CSF of TBM patients, and the predominance of NK cells is associated with a better outcome and survival (143). However, the function of NK cells in TBM patients has not been well addressed. Limited literature has reported the role of NK cells in the inflammatory process related with TBM. NK cells have been demonstrated to be implicated in the pathogenesis of viral and bacterial infections in the CNS. Notably, the activity of NK cells may inhibit herpes virus’s infection (144, 145). The total number of NK cells and CD56^bright^ NK cells in the blood of TBM patients was less than latent TB infection (LTBI) and pulmonary TB (PTB) patients. Cytokines produced by NK cells may contribute to the pathogenic alterations of some CNS bacterial infections (146). Accordingly, the reduction in the total number of NK cells and CD56^bright^ NK cells in the blood of TBM patients may be responsible for the migration of NK cells to the CNS. NK cells in the brain may release proinflammatory cytokines, which in turn induce brain injury in TBM patients. On the other hand, the TBM patients have a higher level of CD56^dim^CD16^+^ NK cells in peripheral circulation. The increased number of CD56^dim^CD16^+^ NK cells in the blood of TBM patients may induce these NK cells to enter the CNS for the purpose of controlling CNS infection. The cytotoxicity induced by NK cells may exert a protective effect for some neurological complications after bacterial infection (147, 148). Meanwhile, van Laarhoven et al. illustrated that the decrease in blood NK cells leads to the enrichment of NK cells in the CNS (143). In addition, they also found that CD69 NK cells reflect the active mobilization of NK cells to the CNS in TBM patients (143). Nevertheless, more information of phenotypical characteristics of NK cells in the CNS during TBM should be studied.

## 7 Interaction of the microglia with CNS macrophages in TBM

Microglia and macrophages are thought to orchestrate the CNS after *M.tb* infection. The intrinsic transcriptional programs, differentiation, and maturation of CNS-associated macrophages (CAMs) and microglia are triggered by environmental factors during the embryonic and postnatal development (149). The CNS is formed earlier than any other organs during embryogenesis with the involvement of immune system to protect the new-born CNS from pathogens (150), and the macrophages exist in the brain during the embryogenesis. Microglia and CAMs are present in the brain parenchyma and perivascular space, respectively (65, 68, 151). Interestingly, foamy macrophages are surrounded by microglia, lymphocytes, epithelioid histiocytes, and new blood vessels and are involved in the formation of CNS granuloma (106, 107). *M.tb* has established a strategy for survival within macrophages by influencing phagosome–lysosome fusion and intervening normal host trafficking events (152, 153). The interaction network between microglia and macrophage mediates the production of cytokines and chemokines in response to *M.tb* invasion (80, 81). Previous studies have shown expression of TNF-α, six different interleukins (IL-1α, IL-1β, IL-6, IL-10, IL-12p40, and IL-18), C-X-C motif chemokine ligands (CXCL8 and CXCL10), matrix metallopeptidases (MMP-1, MMP-3, and MMP-9), granulocyte-macrophage colony-stimulating factor (GM-CSF), macrophage inflammatory protein-1β (MIP-1β), and granulocyte colony-stimulating factor (G-CSF) (67, 80). Lee et al. have revealed that the activation of primary murine microglia in conditioned media from *M.tb*-infected macrophages was associated with the maturation of caspase-1 and IL-1β in microglia during the *M.tb* infection (109). Generally, *M.tb* could be maintained in a silencing status by regulating the expression of interleukins in the homeostatic immune response (154, 155). Moreover, signal transducer and activator of transcription 1 (Stat1) and interferon regulatory factor1 (IRF1) have been verified to be involved in the inflammation response of macrophages and microglia after TBM induced by attenuated *M.tb* (74).

## 8 Interaction of the microglia with astrocytes in TBM

*M.tb* induces both microgliosis and astrogliosis in the CNS, and neurobiological mechanisms are involved in the pathogenesis, which plays a key role in modulating neuronal–glial interactions and synaptic function after *M.tb* infection. The activation of microglia and astrocytes induces neuroimmune interactions in the CNS, producing both pro- and anti-inflammatory cytokines. In addition, the activated microglia and astrocytes have been found in the meningeal exudate (156). Rock et al. have found that dexamethasone, an adjunctive therapy for TBM, could modulate the content of proinflammatory cytokines and chemokines secreted by the CNS macrophages while inhibiting the release of TNF and IL-6 in microglia (80). The astrocyte–microglia lactate shuttle (AMLS) hypothesis states that lactic acid produced by astrocytes through glycolysis in response to the initiation of immune response plays a key role in microglia in the development of TBM (157). Lactic acid is a crucial energy substrate in energy metabolism, and its upregulation contributes to the glucose metabolism in microglia of TBM, thereby performing neuroprotective effects (158, 159).

## 9 Interaction of the macrophages with other cells in the CNS of TBM

Previous studies have found that T lymphocytes and CD4^+^ Th cells play a critical role in granuloma formation. IFN-γ induced by the communication of CD4^+^ Th cells and T lymphocytes activates the macrophages to eliminate the *M.tb* (160–162). Results showed that CD4^+^ and CD8^+^ T cells were gathered around all granuloma types in biopsy specimens, but limited CD4^+^ and CD8^+^ T cells were found in the post-mortem tissues. Moreover, NK cells and monocytes (myeloid mononuclear cells) can kill extracellular *M.tb* and activate a series of signaling pathways including reactive oxygen species (ROS) and glutathione (GSH)-related signaling pathways in macrophages (163). The GSH in NK cells has been reported to be involved in inhibiting the growth of *M.tb*. Thus, exploring the interaction between macrophages and NK cells or lymphocytes may contribute to understanding the infection of the *M.tb* during TBM.

## 10 Conclusions

**Figure 1:**
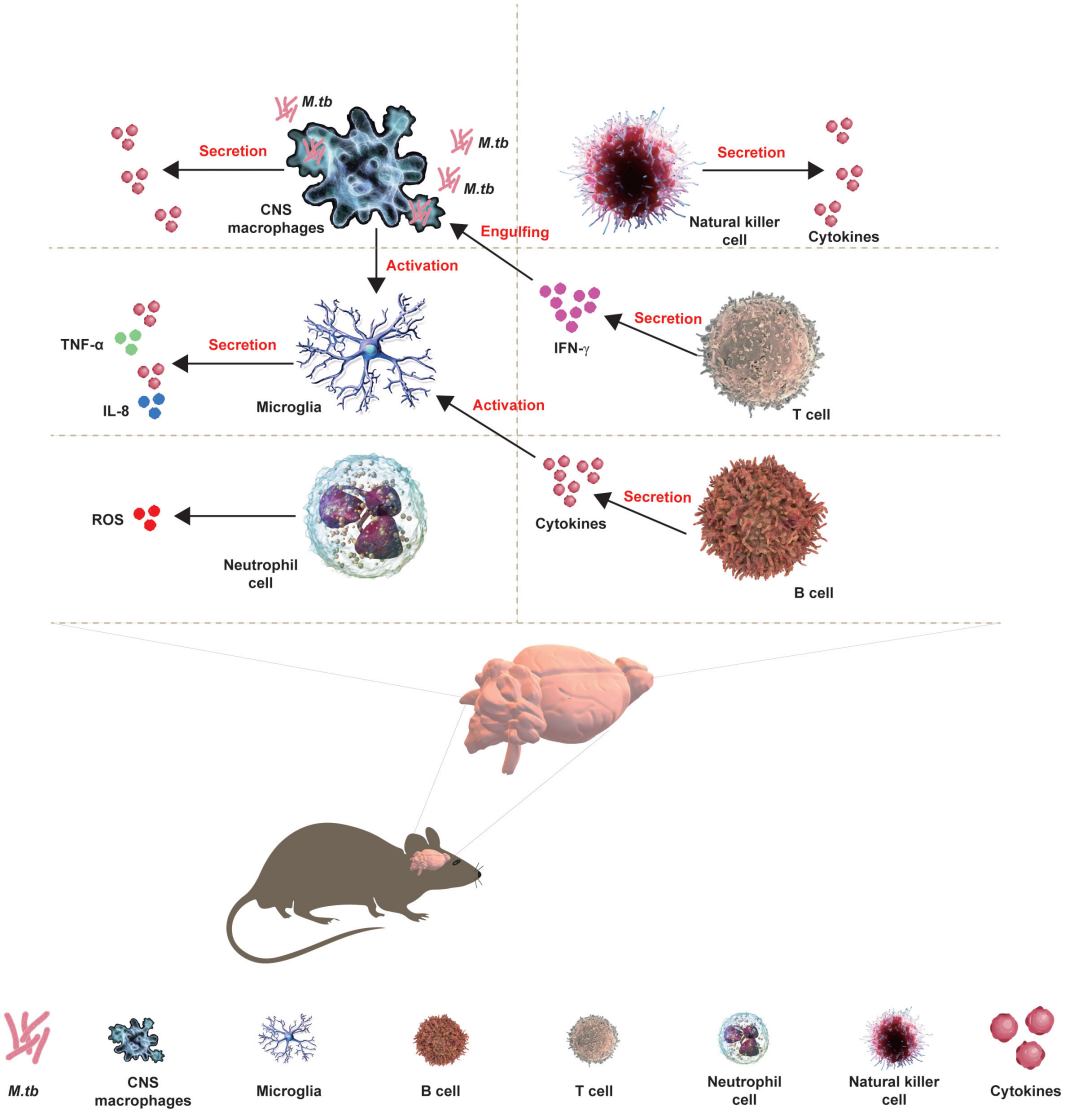
Interaction of CNS with immune cells in TBM. The development of CNS requires the protection of immune cells, including macrophages and microglia, during embryogenesis to ensure the accurate development of CNS and immune response following pathogenic invasion. Microglia are resident macrophages and the major component of the brain’s immune system, working with other immune cells and neurons to maintain the brain’s homeostasis and prevent the invasion of *M.tb*. Moreover, activated T and B lymphocytes have been found in the CSF of TBM patients from the clinical onset of the disease, and more diagnostic tools for TBM measuring the cytokines/chemokines that are secreted by lymphocytes have been developed in clinical practice. Moreover, the interaction of microglia between CNS macrophages or astrocytes has been involved in the TBM.

The development of CNS requires the protection of immune cells, including macrophages and microglia, during embryogenesis to ensure the accurate development of CNS and immune response following pathogenic invasion. Microglia are resident macrophages and the major component of the brain’s immune system, working with other immune cells and neurons to maintain the brain’s homeostasis and prevent the invasion of *M.tb*. Moreover, activated T and B lymphocytes have been found in the CSF of TBM patients from the clinical onset of disease, and more diagnostic tools to measure the cytokines/chemokines secreted by lymphocytes in TBM have been developed in clinical practice. Moreover, the interaction of microglia between CNS macrophages or astrocytes has been involved in TBM (**Figure 1**).

In sum, in the recent work, there remains some doubt about the role of CNS immune cells for TBM. The combination of modern techniques, including single-cell sequencing, multiomics technologies, and deep transcriptomics, will contribute to understanding the role of immune cells of CNS and explore the interaction of immune cells in the development of TBM.

## Author contributions

QM: Conceptualization, Writing – original draft. JC: Writing – original draft. XK: Writing – review & editing. YZ: Writing – review & editing. ZC: Writing – review & editing. HL: Writing – review & editing. LL: Writing – review & editing. SL: Conceptualization, Supervision, Writing – review & editing. XW: Conceptualization, Supervision, Writing – review & editing.
